# Systemic IL-12 Administration Alters Hepatic Dendritic Cell Stimulation Capabilities

**DOI:** 10.1371/journal.pone.0033303

**Published:** 2012-03-13

**Authors:** Tim Chan, Timothy C. Back, Jeffrey J. Subleski, Jonathan M. Weiss, John R. Ortaldo, Robert H. Wiltrout

**Affiliations:** Laboratory of Experimental Immunology, Cancer and Inflammation Program, Center for Cancer Research, National Cancer Institute, National Institutes of Health, Frederick, Maryland, United States of America; Albany Medical College, United States of America

## Abstract

The liver is an immunologically unique organ containing tolerogenic dendritic cells (DC) that maintain an immunosuppressive microenvironment. Although systemic IL-12 administration can improve responses to tumors, the effects of IL-12-based treatments on DC, in particular hepatic DC, remain incompletely understood. In this study, we demonstrate systemic IL-12 administration induces a 2–3 fold increase in conventional, but not plasmacytoid, DC subsets in the liver. Following IL-12 administration, hepatic DC became more phenotypically and functionally mature, resembling the function of splenic DC, but differed as compared to their splenic counterparts in the production of IL-12 following co-stimulation with toll-like receptor (TLR) agonists. Hepatic DCs from IL-12 treated mice acquired enhanced T cell proliferative capabilities similar to levels observed using splenic DCs. Furthermore, IL-12 administration preferentially increased hepatic T cell activation and IFNγ expression in the RENCA mouse model of renal cell carcinoma. Collectively, the data shows systemic IL-12 administration enables hepatic DCs to overcome at least some aspects of the inherently suppressive milieu of the hepatic environment that could have important implications for the design of IL-12-based immunotherapeutic strategies targeting hepatic malignancies and infections.

## Introduction

Dendritic cells (DC) are potent antigen presenting cells (APC) that play important roles in linking innate and adaptive immunity [1], [2], [3]. Although bone marrow-derived and splenic DC have been well characterized in various model systems, the maturation and functions of DC from the liver remains incompletely characterized. The liver is an immunologically unique organ system constantly exposed to antigens that is also capable of maintaining an immunosuppressive, or tolerogenic microenvironment [4], [5], [6], [7]. DC are one of many APC found in the liver that contributes to this tolerogenic phenotype [6], [8]. The liver also contains a higher proportion of plasmacytoid DC (pDC) to conventional DC (cDC) compared to other organs such as the spleen where there is a greater abundance of cDC compared to pDC [5], [9], [10], [11]. Since hepatic pDCs have been implicated in maintaining oral tolerance [12], the overall ratio of cDC:pDC could be very important in the regulation of adaptive immune responses in the liver. Previous studies have shown hepatic DC express lower levels of costimulatory and MHC class II molecules, have reduced cytokine expression and induce minimal T cell proliferation [5], [9], [10], [11], [13]. Taken together, these characteristics of hepatic DC render them a critical component of the inherently immunosuppressive liver microenvironment.

The goal of this study was to investigate whether immunotherapeutic regimens have the potential to overcome the immunosuppressive phenotype of DC in the liver. The reversal of DC from a tolerogenic state to one more closely associated with effector immune responses might hold considerable promise for the treatment of cancers in the liver. One highly promising immunotherapeutic agent is IL-12, a hetero-dimeric cytokine mainly expressed by phagocytes and DC which leads to natural killer (NK) cell activation and the differentiation of T cells towards a T helper 1 (Th1) phenotype, linking innate and adaptive immunity [14], [15]. IL-12 is an attractive candidate, either as a single agent or in combination with other cytokines/immunomodulatory agents, for cancer immunotherapy based upon its potent antitumor effects observed in various preclinical models [16], [17]. Numerous studies have used either systemic or localized IL-12 administration (such as fibroblasts that have been genetically modified to produce IL-12) [18] to further potentiate the immune response and enhance innate and adaptive immunity. However, the effects of systemically administered IL-12 on DC populations, in particular hepatic DC, have not been examined and such studies can be instructive for understanding overall immune function in this unique anatomical compartment.

In this study, we demonstrate that *in vivo* IL-12 administration improves the effector functions of hepatic DC populations to levels which are comparable to splenic DC. Following systemic IL-12 administration, the number of specific hepatic DC subsets increase and are phenotypically and functionally more mature. Furthermore, IL-12 administration enabled hepatic DC to robustly increase T cell activation and proliferation. Our study demonstrates the potential for systemic IL-12 treatment to modulate hepatic DC and suggests the potential for IL-12 based therapies to provide enhanced immune activation in the liver, which is often an immunosuppressive microenvironment.

## Results

### Systemic IL-12 administration expands hepatic and splenic DC

**Figure 1:**
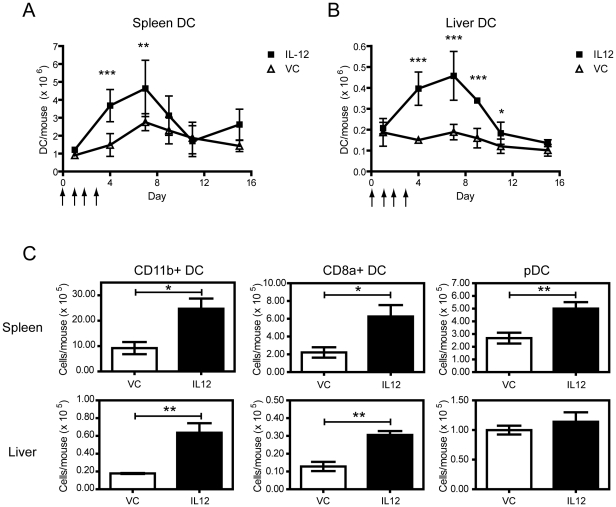
Increased splenic and hepatic DC numbers following systemic IL-12 treatment. BALB/c mice were treated with 1 µg/mouse IL-12 (solid square) or vehicle control (VC; open triangle) for 4 consecutive days from day 0, as indicated with arrows, then leukocyte populations analyzed at various days post treatment. The total DC number (NKp46^−^CD11c^+^ Class II^+^), is shown for the spleen (A) and for the liver (B). The results shown are the mean ± SD from 3–5 mice/group at each time point and representative of 3 separate experiments. (C). Differences in DC subsets were examined in the spleen and liver at day 4 for CD11b^+^ (NKp46^−^CD11c^+^Class II^+^CD11b^+^), CD8α^+^ (NKp46^−^CD11c^+^Class II^+^CD8α^+^), and pDCs (NKp46^−^CD11c^+^Class II^+^B220^+^mPDCA-1^+^SiglecH^+^), respectively. Shown is the mean ± SD from 4–5 mice/group and representative of more than 3 independent experiments. *, *p*<0.05; **, *p*<0.01; ***, *p*<0.001; Mann Whitney U test.

Our previous studies involving systemic administration of IL-12 in BALB/c mice showed repeated daily administration during a cycle of treatment at specific doses is well tolerated [19], [20]. Daily systemic administration of 1 µg IL-12 into BALB/c mice results in an increase in DC (NKp46^−^CD11c^+^Class II^+^) present in the spleen and liver, observed as early as day 4 and reaching a maximum at day 7 (Figure 1A and B, respectively), that gradually declines to homeostatic levels by day 11. Interestingly, the absolute number of hepatic DC remained approximately 1/10^th^ of the absolute number of splenic DC regardless of the treatment. Careful analysis was performed to exclude the confounding NKp46^+^ NK cells, as basal expression levels of CD11c on the NK cells significantly increased following IL-12 treatment (Figure S1) [21], [22]. In addition, reduced bone marrow cellularity was observed following IL-12 administration (data not shown), suggesting the bone marrow as a contributing source for the observed increase in DC.

Next, we analyzed the different subsets of splenic and hepatic DC at day 4 following systemic administration of IL-12. For comparability between DC subset populations in the spleen and liver, the following subsets were analyzed: cDCs broken into CD11b^+^ DC (defined as NKp46^−^CD11c^+^ Class II^+^ CD11b^+^ CD8α^−^; Figure S2), CD8α DC (defined as NKp46^−^ CD11c^+^ Class II^+^ CD11b^−^ CD8α^+^; Figure S2), and pDC (defined as CD11c^int^ Class II^+^ B220^+^ Siglec H^+^ PDCA-1^+^; Figure S3) [9], [13], [23], [24]. The cDC subsets consisting of both the CD11b^+^ and CD8α^+^ DC, were significantly increased in both the spleen and liver (Figure 1C). In the liver, a respective 3.6 fold and 1.8 fold increase in the numbers of CD11b^+^ and CD8α^+^ cDC was observed following IL-12-treatment. On the other hand, the number of hepatic pDC, which comprise the major DC subset in the liver, remained unchanged despite the increase in the number of liver leukocytes following IL-12 administration, in contrast to the increase observed within the spleen (Fig. 1C). Thus, IL-12 treatment increases the ratio of cDC to pDC in the liver while the ratio remains the same in the spleen, despite equivalent increases across the different splenic DC populations. Given that hepatic pDC have been implicated in maintaining oral tolerance [12] and the absolute numbers remain consistent while cDC increase, this suggests IL-12 administration could alter the immunological status of the liver microenvironment.

### IL-12 treatment matures hepatic DC

**Figure 2:**
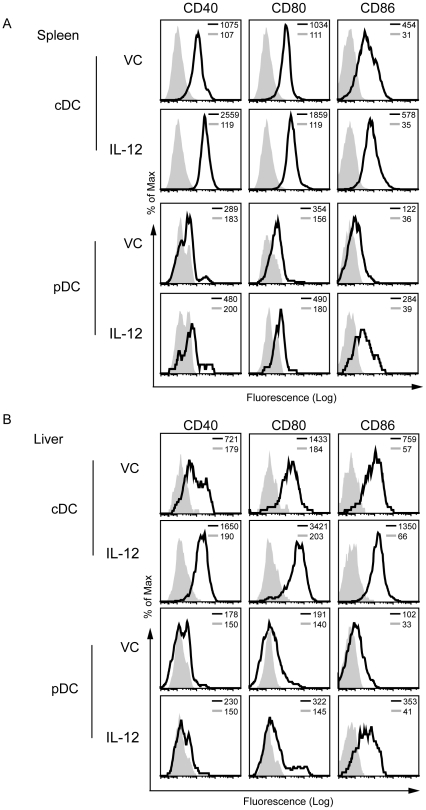
Systemic IL-12 treatment leads to an increase in costimulatory molecule expression on DCs. Mice were treated with one cycle of IL-12 or vehicle control (VC) administration. Leukocytes were harvested the following day from the spleen and liver and the DC populations were analyzed by flow cytometry for expression levels of CD40, CD80 and CD86. The cDC (NKp46^−^CD11c^+^Class II^+^ mPDCA-1^−^ including both CD11b and CD8a DCs) and pDC (NKp46^−^CD11c^+^Class II^+^B220^+^mPDCA-1^+^) in the spleen (A) and liver (B). A representative histogram from one individual mouse is shown with 3–6 mice/group in an experiment that has been repeated in more than 3 independent experiments with similar results. Shaded line is the isotype control and the black solid line is the stain for the respective treatment. The MFI of the corresponding population is shown in the upper right corner of each histogram.

Since systemic IL-12 administration had dramatic effects on DC numbers in the liver, we hypothesized that the maturational state of these DC might also be enhanced. Under basal conditions, the combined population of hepatic DC subsets from vehicle control (VC)-treated mice generally expressed lower basal levels of co-stimulatory molecules compared to their splenic DC counterparts, consistent with the belief that hepatic DCs are in a relatively immature state compared to their splenic counterparts [13], [24]. Following IL-12 therapy, however, the cDC and pDC populations in the spleens (Figure 2A) and livers (Figure 2B) of mice treated with IL-12 displayed significant increases in the cell surface expression of the co-stimulatory molecules CD40, CD80 and CD86, as compared to VC treated mice. It is noteworthy that splenic and hepatic cDCs demonstrated a greater change in the expression pattern of the costimulatory molecules than did splenic and hepatic pDC. Furthermore, studies utilizing SCID mice (which lack T cells but retain NK cells) and IL2Rgamma(c) (γc) knockout mice (which have limited mature T cells and lack NK cells) indicate that the maturation of the DCs seen with systemic IL-12 is partially dependent upon NK cells but not on T cells (Figure S4). Overall, the potential for IL-12 administration to increase the expression of CD40, CD80 and CD86 on hepatic cDC and pDC, to levels similar to or higher than the levels detected on splenic DC subsets, supports the conclusion that IL-12 substantively enhances the cellular maturation of hepatic DC.

### IL-12 treatment reduces antigen uptake and processing in DC

**Figure 3:**
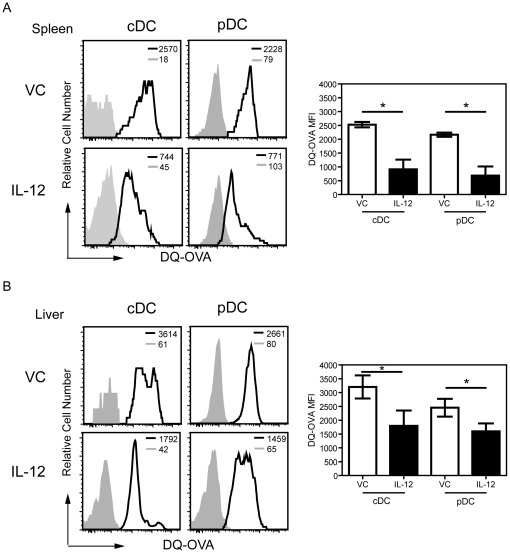
Decreased antigen uptake by splenic and hepatic DCs following systemic IL-12 treatment. Leukocytes from IL-12 or vehicle control treated mice were incubated with 100 µg/ml DQ-OVA for 30 min at 37°C and analyzed by flow cytometric analysis gating on cDC (NKp46^−^CD11c^+^Class II^+^) and pDCs (NKp46^−^CD11c^+^Class II^+^mPDCA-1^+^) populations in the spleen (A) and liver (B). Left hand shows a representative histogram sample for an individual mouse displaying the antigen uptake and processing of DQ-OVA by cDC or pDCs (solid line). The shaded line is the respective isotype control. The MFI of a representative sample is shown in the upper right corner of each histogram. The right side graphically displays the mean ± SD of the DQ-OVA MFI from an independent experiment with 3–5 mice/group that has been repeated 3 times with similar results. *, *p*<0.05; Mann-Whitney U test.

Since antigen uptake by DC is elevated in immature cells and reduced upon cell maturation, we next measured the antigen uptake and processing capabilities of hepatic DC following IL-12 therapy. Using flow cytometry to quantitate fluorescence produced by the uptake and processing of DQ™ Ovalbumin (DQ-OVA), we observed a dramatic decrease in the DQ-OVA MFI after IL-12 administration in both splenic cDC and pDC (Figure 3A). The DQ-OVA MFI in splenic cDC was reduced from 2528±97 to a MFI of 911±351 with IL-12 therapy. Hepatic DC from IL-12 treated mice also displayed similar functional properties of antigen and processing capabilities of DQ-OVA. Following IL-12 treatment, hepatic cDC had a significant reduction in the MFI of DQ-OVA from 3204±417 to 1796±556, indicating DC maturation (Figure 3B). Hepatic pDC from IL-12 administered mice also showed a significant decrease in antigen uptake and processing capabilities of DQ-OVA. Representative contour plots are shown in Figure S5, demonstrating the increased maturation and reduced uptake and processing capabilities of the antigen by co-staining the DC subsets with CD80 following incubation with DQ-OVA. It is also worth noting that the antigen uptake by hepatic DC from VC-treated mice was higher compared to splenic DC from VC-treated mice potentially indicative of the immature state of hepatic DC relative to their splenic counterparts under basal conditions. Collectively, the increased expression of the co-stimulatory molecules and the decreased antigenic uptake by the both cDC and pDC subsets indicate systemic IL-12 therapy results in the maturation of DC, with a substantial net change among hepatic DC.

### *In vivo* administration of IL-12 primes hepatic DC to respond to *ex vivo* TLR agonist stimulation

**Figure 4:**
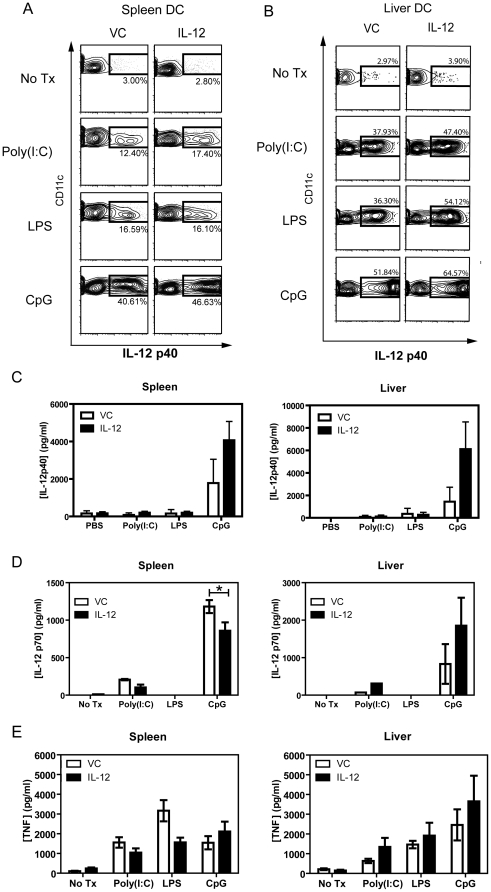
Altered cytokine expression profile of splenic and hepatic DCs. Bulk lymphocytes from the spleen and liver were incubated with media alone (no treatment; no Tx), 25 µg/ml poly(I:C), 1 µg/ml LPS or 2.5 µg/ml CpG for 18 hours. Splenic DC (A) and hepatic DC (B) populations were gated and intracellular IL-12p40 expression examined by flow cytometric analysis. Shown is a representative sample from an individual mouse in an independent experiment repeated 3 times (3–5 mice/group). Purified splenic and hepatic DC pooled from 5–15 mice were cultured under the same conditions and culture supernatants harvested 48 hours later. Cytometric bead array was performed to detect in the supernatants the levels of IL-12p40 (D), IL-12p70 (D) and TNF (E) from splenic DC and hepatic DC. The bar graph displays the mean ± SEM expressed in pg/ml per 10^6^ cells derived from the pooled data obtained from 3 independent experiments. *, *p*<0.05 (Mann Whitney U Test).

DC express a variety of pattern recognition receptors, which include TLRs capable of responding to pathogen-associated molecular patterns. A previous study showed the production of IL-12 by splenic and bone marrow-derived DC initiates a feedback loop by reducing subsequent IL-12 production, thereby limiting the Th1 polarizing immune response [25]. We hypothesized that systemic IL-12 administration may render hepatic DC more responsive to TLR agonist-mediated activation. To test this, bulk leukocytes from the spleen and liver of VC or IL-12-treated mice were stimulated *ex vivo* with either poly(I:C) (TLR3), LPS (TLR4) and CpG (TLR9) and intracellular IL-12 production was analyzed on gated cDC populations. Stimulation of splenic cDC from IL-12 treated mice with the TLR agonists revealed only a modest increase in the percentage of splenic cDC expressing intracellular IL-12p40 (Figure 4A and S6A), whereas significantly increased levels of IL-12p40 were detected in hepatic cDC following stimulation (Figure 4B and S6A). Interestingly, the basal expression levels of IL-12p40 with equivalent TLR-agonist stimulation was higher in VC-treated hepatic cDC compared to splenic cDC (Figure S6A). The expression levels of IL-12p40, as detected by intracellular staining, in the pDC subsets following TLR stimulation was minimal, with the exception of CpG stimulation (data not shown). The detection of intracellular IL-12p40 may reflect the formation of either IL-12p40 homodimers, or heterodimers with the p35 subunit, forming IL-12p70, or with the p19 subunit, forming IL-23. To further delineate between these possibilities, ELISAs were performed to measure total IL-12p40 (Figure 4C) and IL-12p70 (Figure 4D). The levels of IL-23 and IL-27 (p35/EBV-induced gene 3 [EBI3] subunits), as measured by ELISA, in these culture supernatants were below the limits of detection (data not shown). For hepatic DC, the increase in IL-12p40 detected by ICS and ELISA also had a similar trend for increased IL-12p70 expression (Figure 4C). Interestingly, despite differences observed in IL-12 expression levels between splenic and hepatic DC, TNF expression levels remained comparable between systemic IL-12 and VC-treated mice (Figure 4E), although the levels of IL-6 were dramatically elevated in hepatic DCs from TLR-agonist stimulated DCs (Figure S6B). Taken together, these findings demonstrate that exogenous IL-12 administration has the potential to render hepatic DC more responsive to TLR stimulation with altered pro-inflammatory cytokine production that may contribute to enhancing Th1 immune responses.

### IL-12 treatment enhances the T cell proliferative capability of hepatic DC

**Figure 5:**
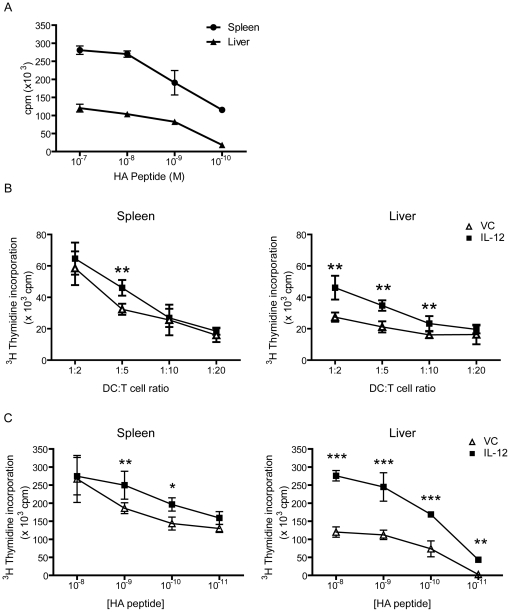
Enhanced T cell proliferation from hepatic DCs following systemic IL-12 treatment. (A) Differential HA TCR proliferation of naïve splenic and hepatic DC. Purified DC were peptide pulsed and incubated with 2×10^4^ HA-TCR T cells at a 1∶1 ratio for 72 hours and 1 µCi of [^3^H]thymidine added during the last 18 hours of culture. (B) Allogeneic mixed lymphocyte reaction. Purified splenic and hepatic DCs from BALB/c mice (H-2^d^) were cultured at differing DC∶T cell ratios with 1×10^5^ purified T cells from C57BL/6 mice (H-2^b^) for 72 hours with 1 µCi of [^3^H]thymidine added during the last 18 hours of culture. (C) HA TCR Tg model. Purified splenic and hepatic DCs were pulsed with varying doses of HA peptide for 2 hours then cultured with 2×10^4^ HA-TCR T cells at a 1∶1 ratio for another 72 hours. 1 µCi of [^3^H]thymidine was added to each well during the last 18 hours of culture. Shown is the mean ± SD done in quadruplicates from one of three independent experiments. *, *p*<0.05; **, *p*<0.01; *** *p*<0.001; Student's T test.

Since our studies outlined above revealed that IL-12 treatment increased DC phenotypic and functional maturation, we sought to determine whether IL-12 treatment could overcome the inherently lower T cell stimulatory capacity of hepatic DC in T cell proliferation assays. As shown in Figure 5A, hemagglutinin (HA)-peptide pulsed splenic DC have at least a 3 fold higher T cell proliferative capability compared to hepatic DC in stimulating responder HA-TCR specific T cells. This data is consistent with previous reports demonstrating substantially reduced stimulatory capacity of hepatic DC compared to their splenic counterparts [13], [26]. In mixed lymphocyte reactions, comparable proliferative responses from naïve C57BL/6 (H-2^b^) responder T cells were observed using allogeneic BALB/c (H-2^d^) stimulator splenic DCs from either IL-12 or VC-treated mice (Figure 5B). Hepatic DC from VC-treated mice showed a 2-fold lower allo-proliferative response compared to their splenic DC counterparts, consistent with the conclusion that hepatic DC have reduced immunostimulatory capabilities [27]. After systemic IL-12 pretreatment, isolated hepatic DC demonstrated greater T cell proliferation capability at the higher stimulator: responder ratios (from 1∶2 to 1∶10 DC∶T cell ratio) compared to hepatic DC from VC-treated mice (Figure 5B). Indeed, the amount of alloreactive T cell proliferation observed with hepatic DC from IL-12 treated mice trended towards the functional stimulatory capacity of splenic DC, on an equivalent per cell basis.

To further determine the effects of IL-12 administration on T cell proliferative capacity, we utilized the transgenic TCR-HA specific mouse model. As shown in Figure 5C, *ex vivo* cultured splenic HA-peptide-pulsed DC from *in vivo* IL-12 treated mice demonstrated improved T cell proliferative responses at various HA peptide concentrations, as compared to VC-treated mice. These results correlated with a similar pattern of T cell proliferation utilizing splenic DC in the allogeneic MLR (Figure 5B). On the other hand, HA peptide-pulsed hepatic DC, containing a mixture of the cDC and pDC subsets, from IL-12 treated mice induced significantly greater T cell proliferation compared to DC from VC-treated mice at all HA peptide concentrations (Figure 5C). An analysis of the specific contributions that each DC subset made to the T cell proliferative response revealed that hepatic pDCs from IL-12 treated mice induced significant fold increase in HA T cell proliferation compared to VC, on an equivalent cell basis (Figure S7). In the spleen, the cDC subset was the likely subset contributing to any enhanced proliferative response seen (Figure S7). The data obtained in this series of T cell proliferation assays indicates that IL-12 therapy may further alter their functional capacity to overcome the reduced proliferative state inherent with hepatic DCs, thus resulting in a greater T cell proliferative response on a per cell basis.

### IL-12 therapy enhances hepatic T cell activation *in vivo* in RENCA-tumor bearing mice

**Figure 6:**
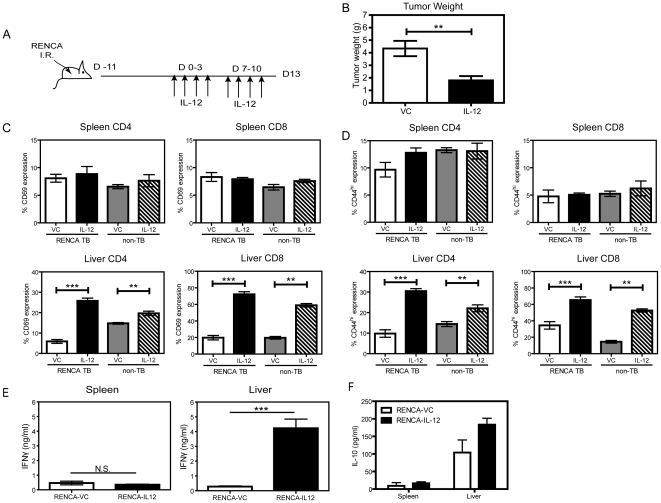
Hepatic T cells are differentially modulated compared to splenic T cells following IL-12 therapy in RENCA tumor bearing mice. (A) Schematic of the treatment cycle for orthotopic RENCA tumor bearing mice, where mice were injected with 1×10^5^ RENCA cells into the kidney capsule then treated with two cycles of IL-12 or VC injections. (B) Mice were euthanized on day 13 following treatment and the weight of the primary tumor was recorded. The activation of CD4 and CD8 T cells (CD3^+^DX5^−^) from the spleen and liver of VC (white) and IL-12 (black) treated RENCA tumor-bearing and VC (grey) and IL-12 (striped) treated non-tumor bearing mice is shown through expression of CD69 (C) and CD44high (D) Shown is the mean ± SEM from two independent experiments with 5 mice/group in each experiment. Purified splenic and hepatic T cells pooled from 2–5 mice/group from RENCA tumor-bearing mice were co-cultured with irradiated RENCA at a 10∶1 ratio for 96 hours followed by IFNγ detection E) and IL-10 (F) in the culture supernatants. The bar graphs represent the means ± SD assayed in triplicate. Shown is a representative experiment that has been repeated twice with similar results. NS not significant, **p<0.01; *** p<0.001; Mann Whitney U test.

The hepatic microenvironment contains a variety of regulatory factors and cells that can dampen local immune responses. Therefore, we next examined whether the phenotypic and functional changes induced by IL-12 in hepatic DCs would also contribute to the *in vivo* modulation of T cell mediated antitumor responses. For these studies, we utilized the RENCA orthotopic murine renal cell carcinoma model whereby the primary tumor readily metastasizes to the lungs, liver and regional lymph nodes. We have previously shown antitumor immunity using IL-12-based treatment regimens that are dependent upon NK and CD8^+^ T cells [19], [28], [29]. For these studies, orthotopic RENCA-bearing mice were treated with 2 cycles of IL-12 as outlined in Figure 6A. Following two cycles of IL-12 treatment, a significant decrease in tumor burden was noted through decreased weight of the primary tumor (Figure 6B), consistent with our earlier results [19], [28]. Equivalent increases in DC numbers were observed in tumor bearing (TB) mice following IL-12 therapy, as compared to naïve, non tumor-bearing (non-TB) mice (data not shown). Interestingly, CD3^+^DX5^-^ T cells obtained from the spleen and liver following IL-12 treatment revealed organ-specific T cell activation patterns. Splenic CD4^+^ and CD8^+^ T cells showed no differences in expression of the early activation marker, CD69, between IL-12 treated and VC-treated mice (Figure 6C). In marked contrast, hepatic CD4^+^ and CD8^+^ T cells had significantly higher CD69 expression in IL-12-treated mice compared to VC-treated mice (Figure 6C). Interestingly, the activation of hepatic T cells was not completely dependent upon the presence of RENCA, because non-TB mice also showed a similar activation of hepatic T cells with IL-12 therapy. Furthermore, while there were no substantial changes in the CD44^hi^ population of splenic CD4^+^ and CD8^+^ T cells detected (Figure 6D), an increased percentage of CD44^hi^ hepatic CD4^+^ and CD8^+^ T cells (Figure 6D) mirrored the CD69 expression pattern (Fig. 6C), and was inversely proportional to the percentage of CD62L^hi^ expressing T cells (data not shown). These findings highlight the potential for IL-12 administration to potently activate hepatic T cells and further contribute to the overcoming of inherent immunosuppression in the liver.

A hallmark function of DC is their ability to induce antigen-specific T cells. To date, no defined tumor antigen has been identified on RENCA cells; therefore, an indirect assay to quantitate IFNγ expression was established using culture supernatants of purified splenic and hepatic T cells co-cultured with irradiated RENCA tumor cells (Figure 6E). Splenic T cells from RENCA-bearing mice treated with IL-12 produced IFNγ to levels similar to T cells obtained from VC treated mice (0.38 ng/ml versus 0.43 ng/ml, respectively). In contrast, the co-culture of purified hepatic T cells from RENCA-bearing mice treated with IL-12 induced 4.2 ng/ml IFNγ as compared to 0.4 ng/ml from hepatic T cells obtained from VC-treated mice. Meanwhile, the expression of IL-10 was minimally induced in the spleen co-cultures while low levels of IL-10 were detected in the hepatic T cell co-cultures, regardless of treatment with IL-12 (Figure 6F). Surprisingly, the treatment with IL-12 further increased the amount of IL-10 produced from the T cells in comparison to the T cells co-cultured from the VC group, but the fold increase was not as substantial as observed for IFNγ production. Taken together, these results show systemic administration of IL-12 specifically enhances the activation of hepatic T cells, but not splenic T cells, which leads to the further development of IFNγ+ T cells in a RENCA tumor-dependent manner.

## Discussion

The liver microenvironment has the distinctive ability to provide seemingly disparate roles in both the formation of a tolerogenic environment that is essential for controlling the development of undesirable immune responses such as orally ingested food antigens, as well as the maintenance of immunoreactive functions frequently associated with lymphoid organs [7], [30]. Alterations to specific hepatic cell populations, induced by therapeutic response of drugs or exposure to pathogenic organisms such as hepatitis virus and tumors, may help to explain some aspects of this complex environment. Resident hepatic DC have been implicated in mediating tolerance and immune suppression within this microenvironment [8], [12], [27]. A more refined understanding of the cellular changes particularly that of hepatic DC compartments, would be valuable for the development of improved strategies that generate more durable adaptive immune responses or the inhibition of potentially detrimental responses in cases of autoimmunity and liver transplantations [31], [32]. In this study, we demonstrate that systemic IL-12 administration not only increased the number of cDC present but more importantly, that these cells were phenotypically and functionally more mature, and more capable of eliciting effective T cell proliferation. The dramatic capability for systemic IL-12 treatment to modify the phenotype and functional activity of hepatic DC to more closely resemble splenic DC, at least under in vitro conditions, thus highlights the potential for IL-12 based therapies for therapy of liver disease.

One of the challenges to studying DC biology in the liver is the relative paucity of resident hepatic DC. Several groups have addressed this issue by expanding the number of DC *in vivo* through the delivery of GM-CSF and/or Flt3-L [24], [26], [33], allowing for the expansion of cDC and pDC, respectively [34]. We opted not to expand the cells within the liver using these methods prior to administering IL-12 as these approaches would likely alter the homeostatic balance of DC and potentially other cells within the local microenvironment [35] and thus not provide representation of the suppressive nature within the liver. Indeed, the homeostatic ratio of cDC∶pDC in each organ may profoundly contribute to the nature of the ensuing immune responses. Since pDC in the liver have been shown to suppress immune responses [12], [13]. the analysis of the cDC∶pDC ratio has become a valuable parameter for the evaluation of the clinical success of patient transplantations [36], [37] and autoimmune disorders [38]. Maintaining the balance of hepatic DC subsets is essential, as exemplified by chronic inflammation and the resulting hepatic fibrosis due to cDC-derived TNFα expression [39]. Under resting conditions, the homeostatic ratio of cDC∶pDC in the spleen is relatively high in dramatic contrast to the liver where we show pDC to comprise the major DC subset.. Following IL-12 treatment, however, we demonstrated the cDC∶pDC ratio within the liver rapidly increased as a result of significant cDC expansion and eventually decreased following the cessation of IL-12 administration. The cDC∶pDC ratio in the spleen, however, remained relatively unaffected by IL-12 treatment. The IL-12 mediated increase in cDC∶pDC ratio of the liver may thus contextually provide the appropriate conditions to mount more robust immune responses as increased DC maturation was also observed.

IL-12 binds to the complex of IL12Rb1 and IL12Rb2 subunits expressed on T cells and NK cells [14]. DC have also been shown to express both of these subunits [40], [41]. The maturation of the DCs which we describe may therefore be due to direct interactions between the administered IL-12 and its receptor expressed on the DC or through indirect mechanisms. In the latter regard, it has been reported that maturation of hepatic DC were dependent upon the activation of NKT cells through either administration of alpha-galactosylceramide (αGalCer) or through infection with MCMV [11]; however, mature DC, based upon co-stimulatory expression, were still observed following IL-12 administration to CD1d knock-out mice, which developmentally lack NKT cells (our unpublished observations). On the other hand, treating IL2Rgamma(c) mice, which lack NK and mature T cells, with systemic IL-2 did not result in DC maturation in the spleen and liver, whereas DC maturation was observed with IL-12 administration in SCID mice, consistent with the observations seen in wild-type mice (Figure S4). The reciprocal crosstalk communication between NK and DCs has been the subject of considerable investigations [42], [43], [44], and we surmise that cellular interactions may play a significant role in the DC activation phenotype observed. Studies involving the selective transient depletion of cDC in CD11c-diphtheria toxin receptor (CD11c-DTR) mice [45] mice, which express the diphtheria toxin receptor and GFP in CD11c^hi^ cells, may provide detailed mechanism in some instances. Unfortunately, the determination of the contributing cellular effects *in vivo* with IL-12 therapy would likely be confounded by the promiscuous expression of CD11c in activated NK cells following IL-12 treatment. In this instance, a population of the NK cells would become sensitive to diphtheria toxin-mediated elimination, by means of non-specific expression of the diphtheria toxin receptor and GFP on NK cells following IL-12 treatment (Figure S1 and our unpublished observations).

The liver has been classically thought to harbor dying T cells as a mechanism for removing them from the lymphatic system. Crispe and colleagues however demonstrated that activated T cells within the liver remain functional, expand and secrete IFNγ and potentially mediate effector activities in lymphoid organs [46], [47]. It is currently unknown whether the activated T cells observed in our study are derived from systemically activated T cells that subsequently become trapped in the liver [48] or if these represent intrahepatic-derived T cells [49]. Supporting the latter point, hepatic DC from IL-12-treated mice were capable of inducing strong T cell proliferation *in vitro*, similar to that seen with splenic DC, on a per cell basis. Our data demonstrates that systemic IL-12 administration alters the hepatic DC subset composition and induces DC maturation, in turn resulting in further T cell priming of naïve cells. Further supporting this, a significant proportion of activated hepatic T cells, based upon expression of CD69 and CD44^hi^, were present in both RENCA-tumor and non-tumor bearing mice and high levels of IFNγ were detected in the supernatants of hepatic T cell from RENCA tumor-bearing mice co-cultured with RENCA cells compared splenic T cells under similar conditions. Based upon these collective findings, future studies utilizing a hepatocellular tumor model system may provide an opportunity to assess the contributing role that DC have in altering the T cell responses in the hepatic microenvironment during tumor development and progression. Additionally, further studies on the fate and function of the activated T cells after cessation of IL-12 within the liver may provide additional novel insight into the design of localized therapeutic strategies to maximize immune responses against intra-hepatic tumors or infectious pathogens that target the liver.

Overall, our findings demonstrate that systemic IL-12 administration alters the balance of hepatic DC favoring the generation of an immune response. Splenic DC have an inherently greater stimulatory capacity and maturational state compared to resident hepatic DC. Following systemic IL-12 administration, hepatic DC become more mature and acquire a significantly greater capacity to stimulate T cells and increased cytokine production as compared to their splenic DC counterparts. Collectively, IL-12 treatment alters the hepatic microenvironment by enhancing the function of resident hepatic DC and provisionally providing an opportunity to overcome a component of the inherently suppressive milieu of the hepatic microenvironment. Our data suggests that modifying hepatic DC function might be further exploited with biological response modifiers as a mean to develop new strategies, either systemically or locally, for targeting hepatic malignancies and infections.

## Materials and Methods

### Animals

BALB/c and C57BL/6 mice (6–12 weeks old) were obtained from Jackson Laboratories (Bar Harbor, ME). The T cell receptor HA transgenic mice, specific for peptide 518–526 of HA, were a gift from Dr. Tom Sayers (NCI/NIH), and originally obtained from Dr. Linda Sherman (The Scripps Research Institute, La Jolla, CA). SCID and IL2Rgamma(c) mice were generously provided by Drs Howard Young (NCI/NIH) and Dan McVicar (NCI/NIH), respectively. All mice were housed under pathogen-free conditions in animal facilities located at the NCI (Frederick MD), following the guidelines in accordance with protocols (#07-068 and #10-256) approved by the NCI-Frederick Animal Care and Use Committee.

### Treatment protocol

Recombinant murine IL-12 was from Peprotech (Rocky Hill, NJ). Mice were injected intraperitoneally (i.p.) for one cycle, unless otherwise stated, with either vehicle control (VC; HBSS containing 0.1% normal mouse serum) or with 1 µg IL-12 for 4 consecutive days. Mice were euthanized between days 4 and 7 after the start of treatment, unless otherwise stated, and the livers and spleens harvested.

### Liver and spleen leukocyte isolation

Liver leukocytes were isolated as previously described [28] with a few modifications. After flushing with HBSS, the liver was minced and incubated with 1 mg/ml Collagenase D (Roche Diagnostics, Indianapolis, IN) in RPMI media (Mediatech, Manassas, VA) at 37°C for 30 min then further disrupted using a Stomacher 80 (Seward, West Sussex, United Kingdom) system in HBSS supplemented with 2 mM EDTA and 0.1% BSA. Liver nonparenchymal cells were collected by centrifugation at 325× *g* for 10 min at 4°C, washed with cold HBSS, and centrifuged. The cell pellet was resuspended in 40% Percoll (Amersham Pharmacia, Piscataway, NJ) then underlaid with 80% Percoll and centrifuged for 25 min at 850× *g*. All Percoll solutions were prepared as 1× DPBS/Percoll diluted with DMEM (Mediatech). Leukocytes were collected at the interface and washed twice in HBSS.

Spleens were incubated in Collagenase D (1 mg/ml) for 30 min at 37°C. Splenocytes were prepared by mechanical disruption followed by treatment with ACK lysis buffer (Invitrogen, Grand Island, NY). Leukocyte counts were obtained using a Sysmex KX-21 analyzer (Mundelein, IL). Splenic and hepatic DC were isolated using DX5 microbeads (Miltenyi Biotec, Auburn, CA) for NK cell depletion followed by positive selection of DC using CD11c microbeads according to manufacturer's protocol (Miltenyi Biotec). DC were further purified by sorting on a FACSAria (Becton Dickinson) with >95% purity.

### Analysis of cell phenotype by flow cytometry

All antibodies utilized were purchased from either BD Biosciences (San Jose, CA) or eBiosciences (San Diego, CA). The following fluorescently-labeled or biotinylated antibodies were utilized: CD11c (HL3, N418), MHC Class II (M5/114.15.2), CD40 (HM40-3), CD80 (16-10A1), CD86 (GL1), B220 (RA3-6B2), CD11b (M1/70), CD49b (DX5), Siglec H (eBio440c), PDCA-1 (eBio927), NKp46 (29A1.4), CD44 (IM7) and CD69 (MEL-14). All samples were incubated 30 min on ice with 2.4G2 ascites fluid to block Fcγ receptor binding before adding fluorochrome-conjugated antibodies. Flow cytometric data was collected on a BD FACSort and LSRII flow cytometers (BD Biosciences) and analyzed using FlowJo 7.5 software (Treestar; Ashland, OR).

### Cytokine expression

Bulk splenic and hepatic leukocytes were prepared as described above and cells were cultured in complete RPMI media. Cells were stimulated with either 25 µg/ml poly(I:C) (Sigma; St. Louis, MO), 1 µg/ml LPS (Sigma) or 2.5 µg/ml CpG, containing an equimolar combination of phosphorothiate ODNs 1555 (GCTAGACGTTAGCGT) and 1466 (TCAACGTTGA), a kind gift from Dr. Dennis Klinman, (NCI/NIH) [50] and incubated for 12 h under standard conditions with 1 µl/ml GolgiPlug (BD Biosciences) added to the cultures for the last 10 h. Cells were harvested and Fcγ receptors were blocked using 2.4G2 prior to staining for specific DC populations. Cells were washed then fixed with BD Cytofix/Perm solution followed by intracellular staining with IL-12p40 (C15.6) and TNFα (MP6-XT22) antibodies in BD Perm/Wash solutions. Purified DC were obtained according to manufacturer's protocol (Miltenyi Biotec) and cultured in media containing 20 ng/ml GM-CSF (Biosource; Camarillo, CA) with the TLR agonists indicated above. Culture supernatants were harvested 48 h later and analyzed for cytokine expression using a proinflammatory cytometric bead array (CBA; BD Biosciences) on a BD FACScan cytometer (BD Biosciences) or by standard ELISAs (eBioscience).

### Antigen uptake assays

Isolated leukocytes were incubated for 30 minutes at 37°C with 100 µg/ml of DQ™ Ovalbumin (DQ-OVA; Molecular Probes, Eugene, OR) in RPMI media. Cells were harvested, washed four times with ice-cold HBSS then stained and gated on specific DC populations by flow cytometry analysis. Cells that uptake and process antigen were determined by green fluorescence occurring when DQ-OVA undergoes proteolytic degradation during antigen processing.

### T cell proliferation assays

Mixed allogeneic reactions were performed using varying doses of irradiated BALB/c splenic and hepatic DCs (3000 rads) cocultured with 10^5^ purified T cells from C57BL/6 mice using Pan T cell microbeads (Miltenyi Biotec). The HA-specific T cell culturing conditions utilized a 1∶1 ratio of irradiated DC to purified HA T cells. DCs were pulsed with the HA peptide (IYSTVASSL) generously provided by Dr. Tom Sayers, (NCI/NIH) [51] at varying doses for 2 h then co-cultured with purified HA-TCR T cells. Co-cultures were incubated at 37°C for 72 h in 96 well U-bottom plates (Costar; Corning, NY), with 1 µCi [^3^H]thymidine/well added during the final 18 h of culture. Cells were harvested onto a filtermat and the amount of ^3^H incorporation was determined using a Wallac 1450 microbeta counter (Perkin Elmer). All counts per minute (cpm) displayed were corrected by subtracting the incorporation amount from media alone. Background values, of T cells only, were below 3000 cpm.

### Tumor model and assaying T cell function

BALB/c mice were injected orthotopically into the kidney capsule with 10^5^ RENCA cells [19]. After 11 days, mice were treated with two cycles of IL-12 treatment with a 3 day rest between cycles. Mice were euthanized after 2 cycles of IL-12 and the primary tumors were measured and weighed. T cells from livers and spleens were phenotyped by flow cytometric analysis. T cells obtained from the spleen and liver were column purified using a negative selection method (Miltenyi), then co-cultured with irradiated RENCA cells (12,000 rads) at a ratio of 10∶1 in complete RPMI media. Culture supernatants were harvested 96 h later and IFNγ expression levels were measured by Cytometric Bead Array (BD Biosciences).

### Statistics

Data were analyzed using Prism 5 (GraphPad Software Inc; La Jolla, CA) utilizing the Student's t test or the non-parametric Mann-Whitney U-test where appropriate. P values<0.05 were considered statistically significant. Graphs show the mean ± SD or SEM, as stated in the figure legend.

## Supporting Information

Figure S1 **Increased expression of CD11c on NK cells following IL-12 treatment.** Mice were injected with VC or IL-12 (1 mg/mouse) for four consecutive days. Flow cytometric analysis was performed on day 5 to evaluate CD11c expression on the gated NKp46+ cells in the spleen and liver following IL-12 treatment. Percentage of CD11c expressing NK cells is indicated in the upper right hand corner of each histogram. Shaded line represents the isotype control.
(TIF)

Figure S2 **Phenotypic characterization of cell surface markers distinguishing CD11b+ and CD8a+ DCs in the spleen and liver.** Splenic and hepatic leukocytes were stained with a panel of antibodies to gate DC specific populations by flow cytometry. DCs were determined by gating on NKp46− CD11c+ Class II+ cells then further categorized into CD11b+ and CD8a+ DCs, based upon expression of CD11b and CD8a markers, respectively. This gating strategy was utilized to enumerate specific DC subsets.
(TIF)

Figure S3 **Phenotypic characterization of cell surface markers characterizing splenic and hepatic pDCs.** Splenic and hepatic leukocytes were stained with a panel of antibodies to gate the pDC population by flow cytometry. The pDCs were defined based upon NKp46− CD11c+ Class II+ B220+ mPDCA-1+ and Siglec H+ expression. This gating strategy was utilized to enumerate pDC subsets.
(TIF)

Figure S4 **Hepatic DC maturation is dependent upon the presence of NK cells.** Mice were treated with either IL-12 (1 mg/mouse) or VC i.p. for four consecutive days in C57BL/6, SCID (which lack T cells but have NK cells present), and IL2Rgamma(c) KO mice (which lack both T and NK cells). Multi-color flow cyotmetric analysis was performed on gated hepatic cDC populations. A representative histogram is shown from an independent experiment with 2–6 mice per group and repeated 3 times. In the histogram overlays, the shaded line is the isotype control, dashed line is the vehicle control and the solid line is with IL-12 administration.
(TIF)

Figure S5 **Co-staining of splenic and hepatic DC subsets for antigen processing with costimulatory molecule expression.** Bulk lymphocytes from the spleen and liver were incubated with DQ-OVA, as indicated in Material and Methods, then co-stained with anti-CD80 antibody for flow cytometric analysis. Shown are representative contour plots from DC subsets from the spleen and liver from 4–8 mice/group. This has been repeated in two independent experiments.
(TIF)

Figure S6 **Altered cytokine expression from splenic and hepatic DCs following TLR stimulation.** Bulk lymphocytes from the spleen and liver were incubated with media alone (no treatment; no Tx), 25 mg/ml poly(I:C), 1 mg/ml LPS or 2.5 mg/ml CpG for 18 hours. (A) Splenic DC and hepatic DC populations were gated and intracellular IL-12p40 expression examined by flow cytometric analysis. The graph shows the mean ± SEM of DC expressing IL-12p40 derived from 3 independent experiments with 3–5 mice/group. * p<0.05; Mann Whitney U test. (B) Culture supernatants were collected from similar TLR-stimulated DC cultures after 48 hours to detect IL-6 expression.
(TIF)

Figure S7 **Enhanced HA T cell proliferation of specific splenic and hepatic DC subsets following IL-12 treatment.** FACS-sorted purified DC populations cDC and pDC were pulsed with 10–10 M HA peptide then cocultured for 72 hrs with equal numbers of Cln-4 HA purified T cells in quadruplicates. Cultures were pulsed with 1 mCi of 3H per well 18 hrs prior to harvesting the cells onto filter mats to quantitate cell proliferation. Data shown is expressed as HA stimulation index calculated as the ratio of Cln-4 T cell proliferation in the presence of HA peptide-pulsed DC to Cln-4 T cells only. Shown is the mean +/− S.D.; *** p<0.001, Student T test.
(TIF)
