# Supplementary figures and images for: Systemic IL-12 Administration Alters Hepatic Dendritic Cell Stimulation Capabilities
Source: PLoS One. 2012 Mar 13;7(3):e33303. doi: 10.1371/journal.pone.0033303 (PMC3302816; doi:10.1371/journal.pone.0033303)

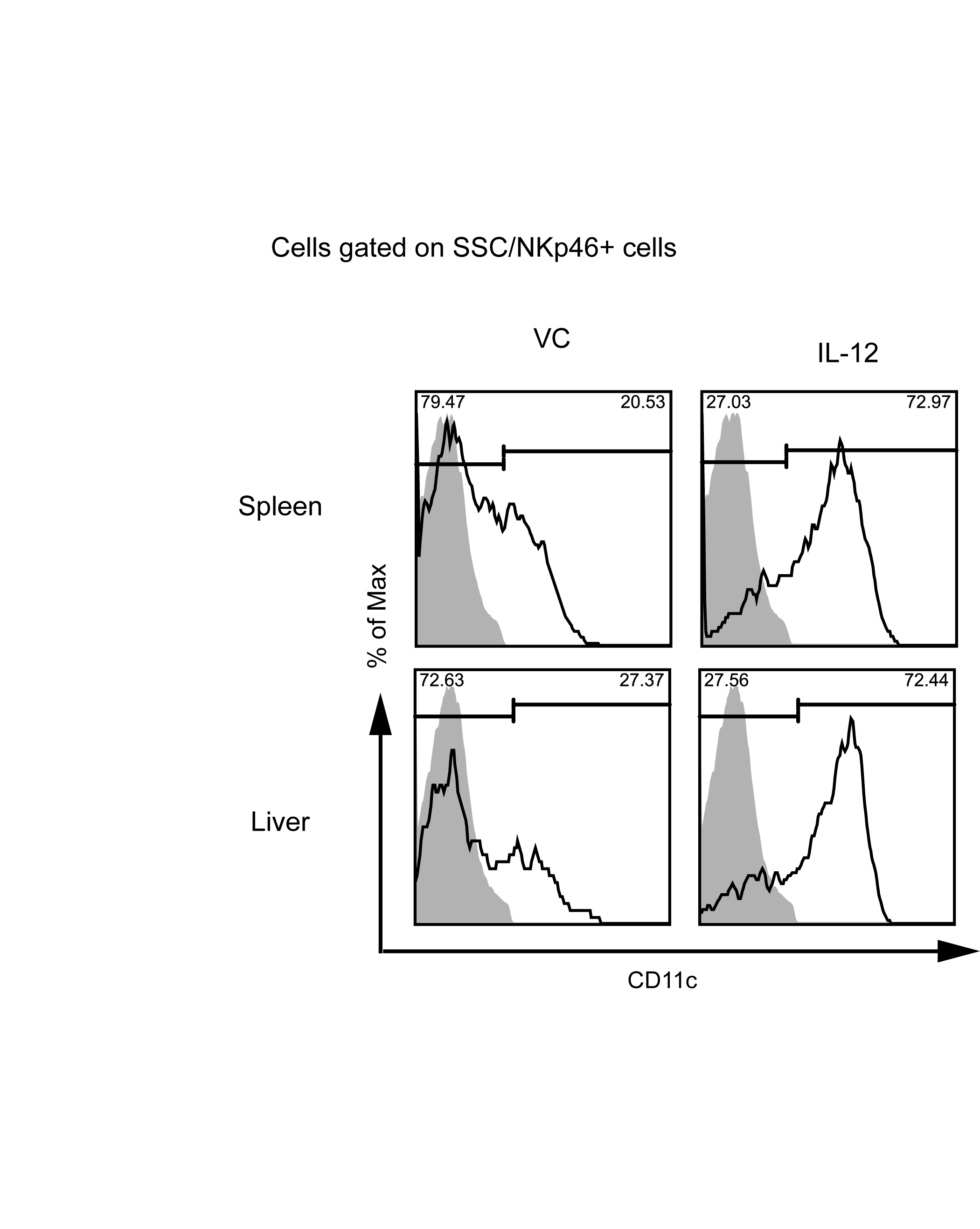

Supplement: Figure S1 — Increased expression of CD11c on NK cells following IL-12 treatment. Mice were injected with VC or IL-12 (1 mg/mouse) for four consecutive days. Flow cytometric analysis was performed on day 5 to evaluate CD11c expression on the gated NKp46+ cells in the spleen and liver following IL-12 treatment. Percentage of CD11c expressing NK cells is indicated in the upper right hand corner of each histogram. Shaded line represents the isotype control. (TIF) [file pone.0033303.s001.tif]

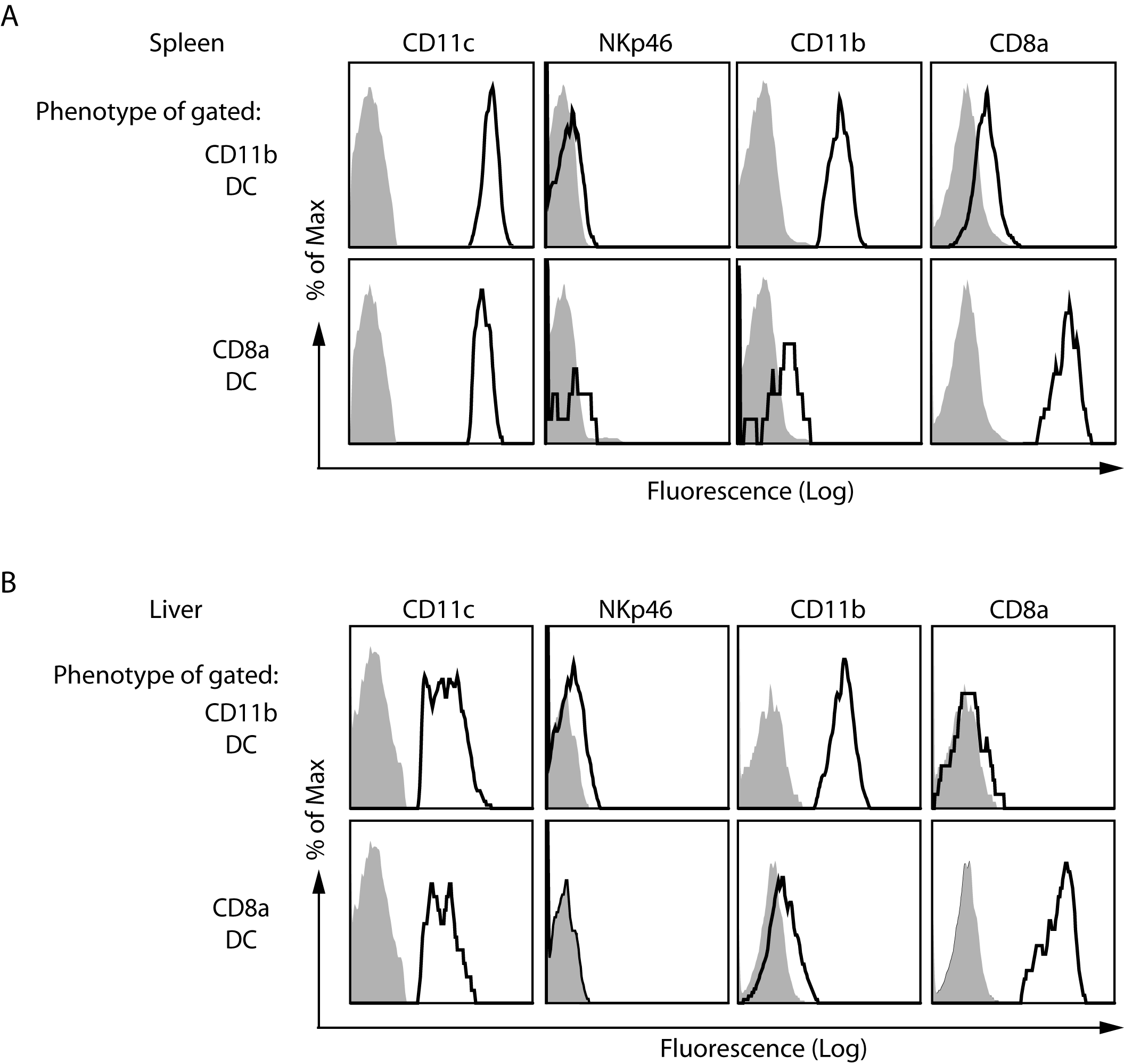

Supplement: Figure S2 — Phenotypic characterization of cell surface markers distinguishing CD11b+ and CD8a+ DCs in the spleen and liver. Splenic and hepatic leukocytes were stained with a panel of antibodies to gate DC specific populations by flow cytometry. DCs were determined by gating on NKp46− CD11c+ Class II+ cells then further categorized into CD11b+ and CD8a+ DCs, based upon expression of CD11b and CD8a markers, respectively. This gating strategy was utilized to enumerate specific DC subsets. (TIF) [file pone.0033303.s002.tif]

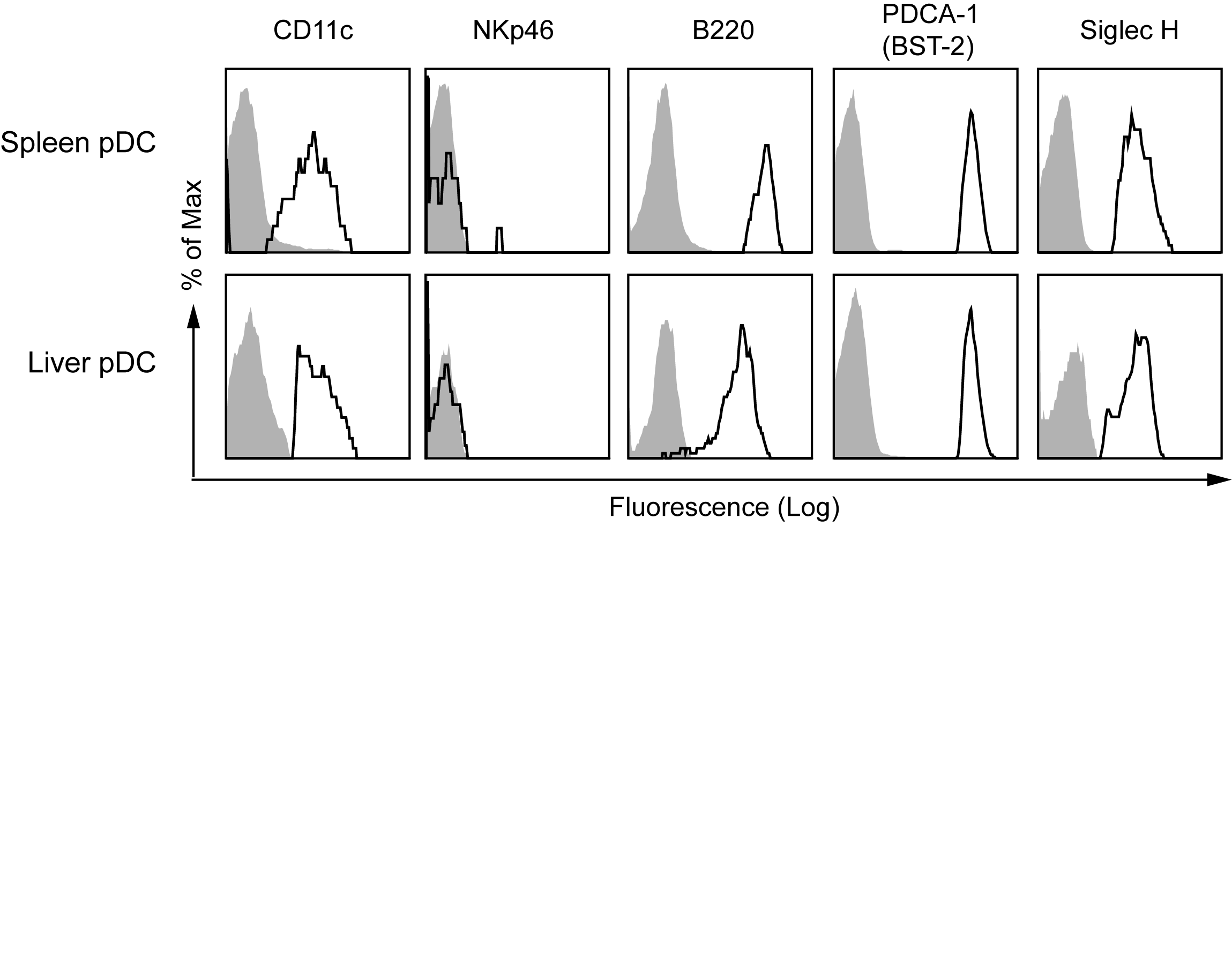

Supplement: Figure S3 — Phenotypic characterization of cell surface markers characterizing splenic and hepatic pDCs. Splenic and hepatic leukocytes were stained with a panel of antibodies to gate the pDC population by flow cytometry. The pDCs were defined based upon NKp46− CD11c+ Class II+ B220+ mPDCA-1+ and Siglec H+ expression. This gating strategy was utilized to enumerate pDC subsets. (TIF) [file pone.0033303.s003.tif]

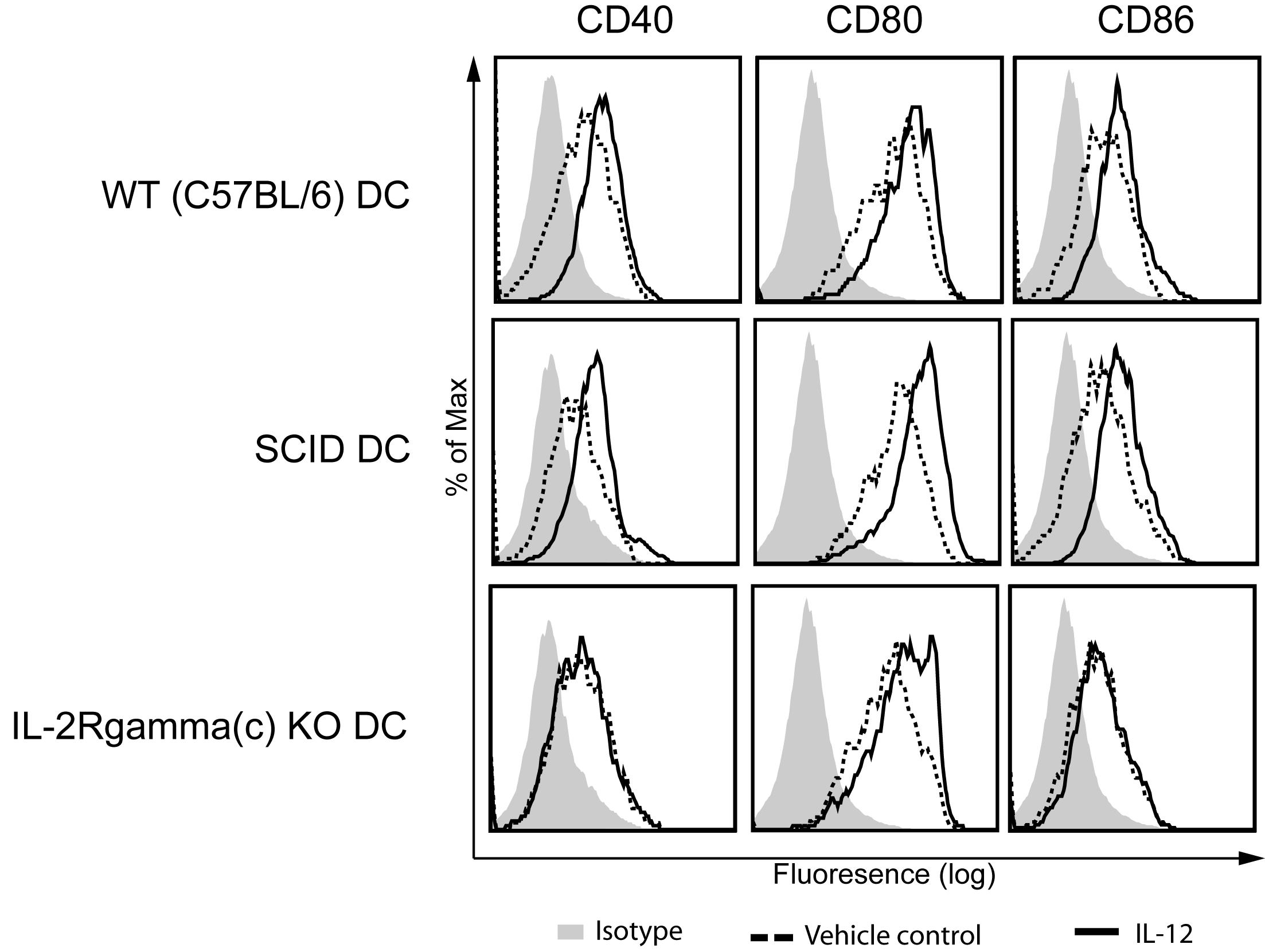

Supplement: Figure S4 — Hepatic DC maturation is dependent upon the presence of NK cells. Mice were treated with either IL-12 (1 mg/mouse) or VC i.p. for four consecutive days in C57BL/6, SCID (which lack T cells but have NK cells present), and IL2Rgamma(c) KO mice (which lack both T and NK cells). Multi-color flow cyotmetric analysis was performed on gated hepatic cDC populations. A representative histogram is shown from an independent experiment with 2–6 mice per group and repeated 3 times. In the histogram overlays, the shaded line is the isotype control, dashed line is the vehicle control and the solid line is with IL-12 administration. (TIF) [file pone.0033303.s004.tif]

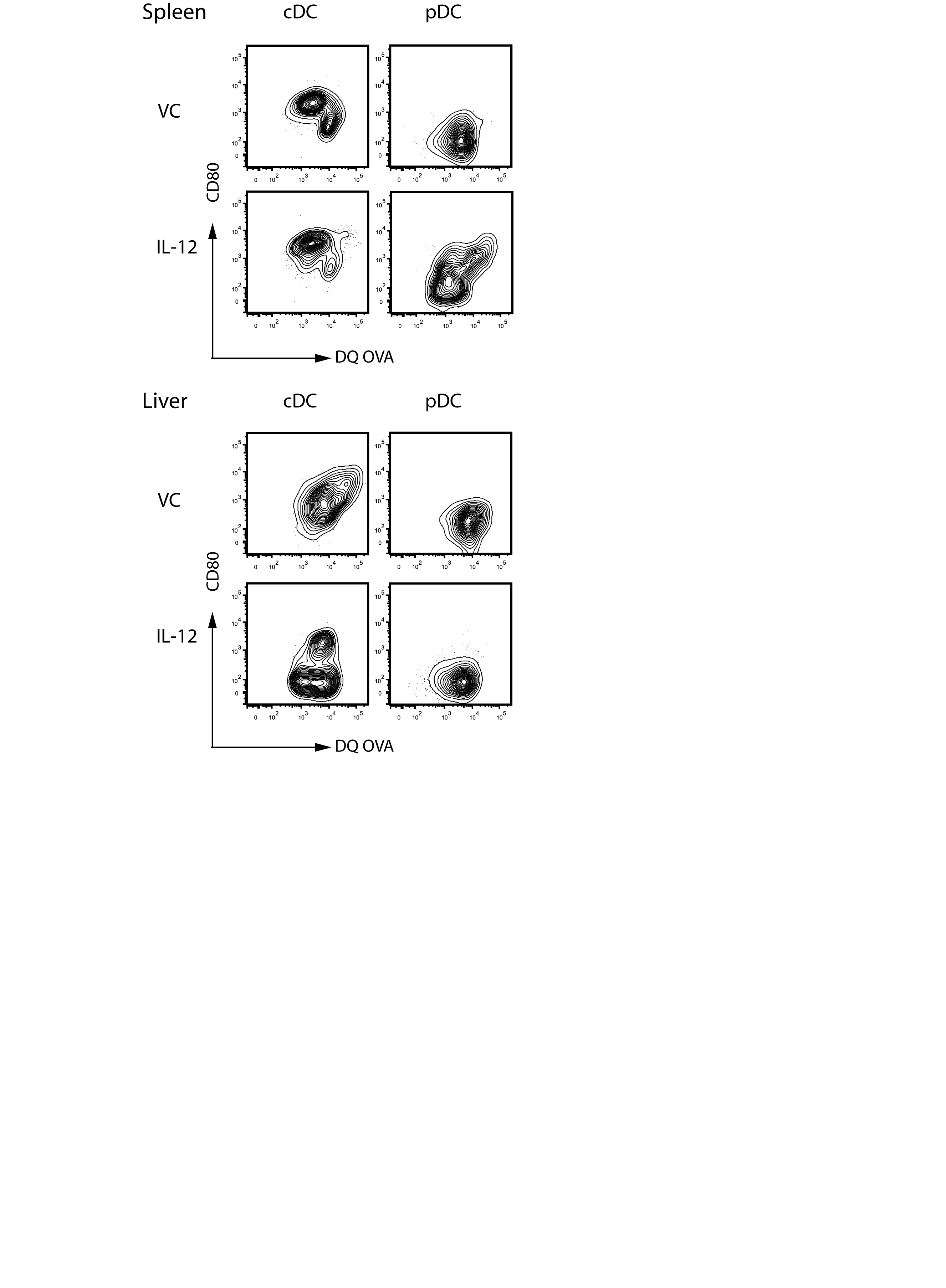

Supplement: Figure S5 — Co-staining of splenic and hepatic DC subsets for antigen processing with costimulatory molecule expression. Bulk lymphocytes from the spleen and liver were incubated with DQ-OVA, as indicated in Material and Methods, then co-stained with anti-CD80 antibody for flow cytometric analysis. Shown are representative contour plots from DC subsets from the spleen and liver from 4–8 mice/group. This has been repeated in two independent experiments. (TIF) [file pone.0033303.s005.tif]

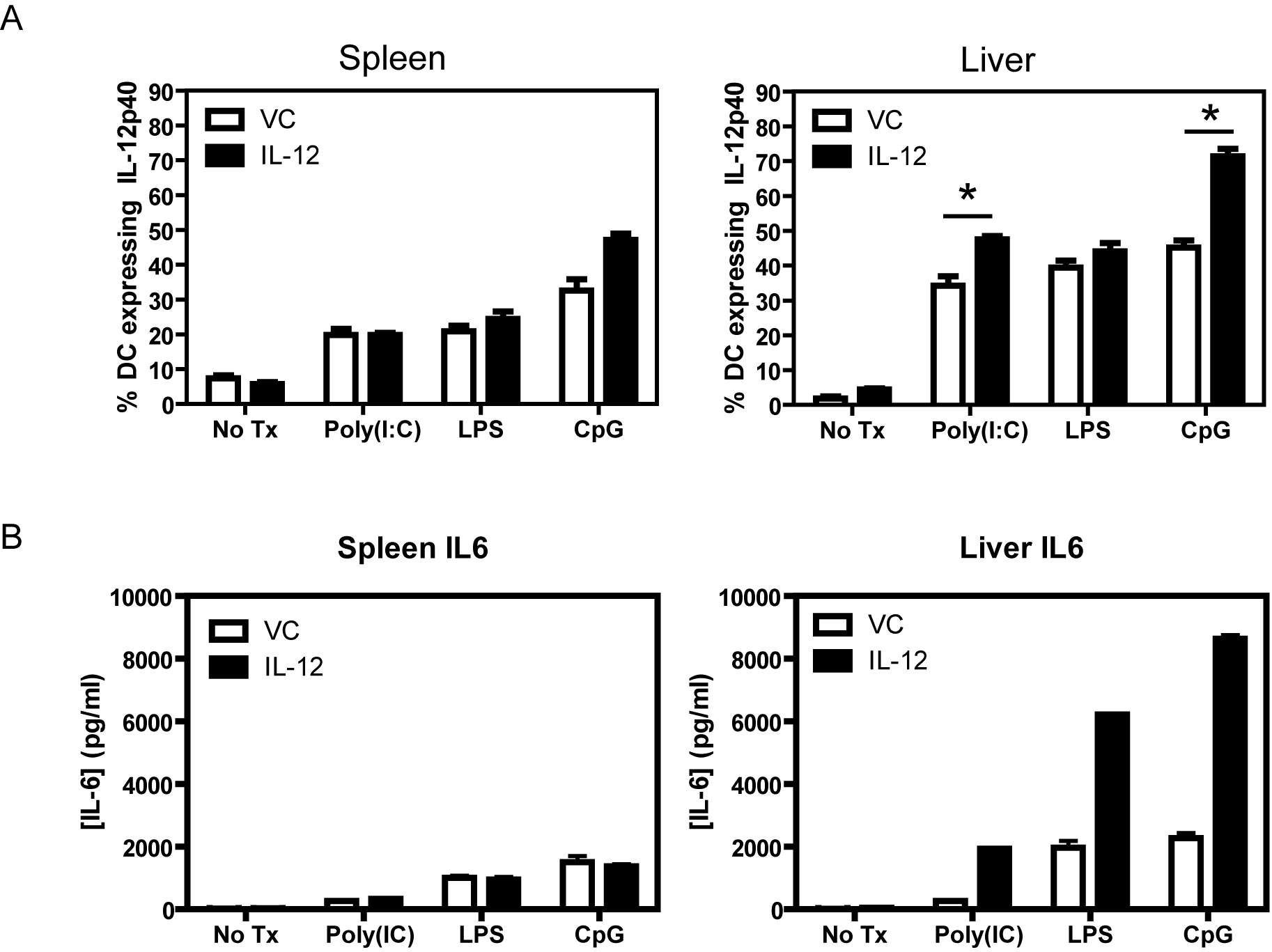

Supplement: Figure S6 — Altered cytokine expression from splenic and hepatic DCs following TLR stimulation. Bulk lymphocytes from the spleen and liver were incubated with media alone (no treatment; no Tx), 25 mg/ml poly(I:C), 1 mg/ml LPS or 2.5 mg/ml CpG for 18 hours. (A) Splenic DC and hepatic DC populations were gated and intracellular IL-12p40 expression examined by flow cytometric analysis. The graph shows the mean ± SEM of DC expressing IL-12p40 derived from 3 independent experiments with 3–5 mice/group. * p<0.05; Mann Whitney U test. (B) Culture supernatants were collected from similar TLR-stimulated DC cultures after 48 hours to detect IL-6 expression. (TIF) [file pone.0033303.s006.tif]

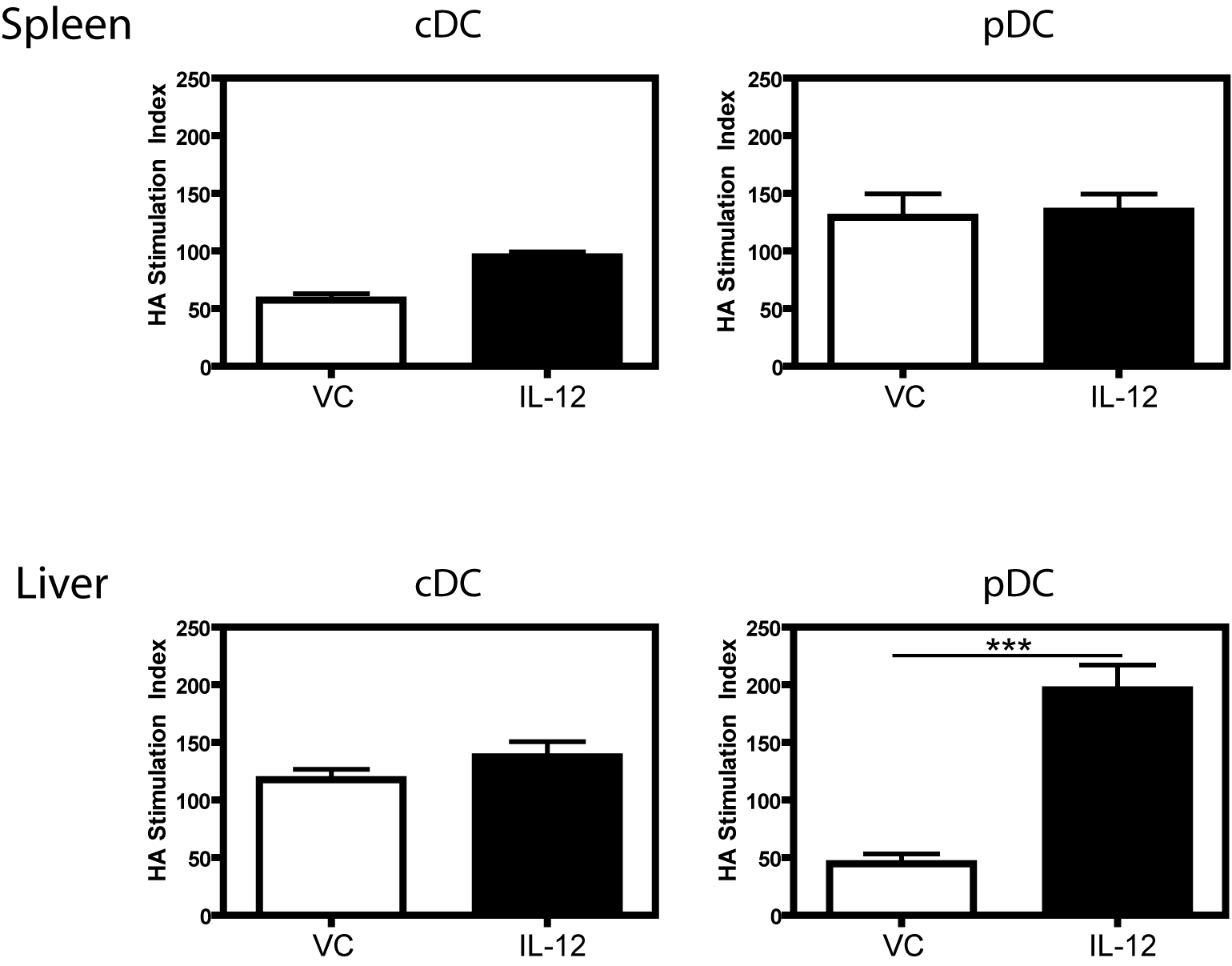

Supplement: Figure S7 — Enhanced HA T cell proliferation of specific splenic and hepatic DC subsets following IL-12 treatment. FACS-sorted purified DC populations cDC and pDC were pulsed with 10–10 M HA peptide then cocultured for 72 hrs with equal numbers of Cln-4 HA purified T cells in quadruplicates. Cultures were pulsed with 1 mCi of 3H per well 18 hrs prior to harvesting the cells onto filter mats to quantitate cell proliferation. Data shown is expressed as HA stimulation index calculated as the ratio of Cln-4 T cell proliferation in the presence of HA peptide-pulsed DC to Cln-4 T cells only. Shown is the mean +/− S.D.; *** p<0.001, Student T test. (TIF) [file pone.0033303.s007.tif]
